# Functional Effect of the Mutations Similar to the Cleavage during Platelet Activation at Integrin β3 Cytoplasmic Tail when Expressed in Mouse Platelets

**DOI:** 10.1371/journal.pone.0166136

**Published:** 2016-11-16

**Authors:** Xiaofeng Shi, Jichun Yang, Xiongying Cui, Jiansong Huang, Zhangbiao Long, Yulan Zhou, Ping Liu, Lanlan Tao, Zheng Ruan, Bing Xiao, Wei Zhang, Dongya Li, Kesheng Dai, Jianhua Mao, Xiaodong Xi

**Affiliations:** 1 State Key Laboratory of Medical Genomics, Shanghai Institute of Hematology, Collaborative Innovation Center of Hematology, Sino-French Research Center for Life Sciences and Genomics, Ruijin Hospital, Shanghai Jiao Tong University School of Medicine, Shanghai, 200025, China; 2 Department of Hematology, Affiliated Hospital of Jiangsu University, Zhenjiang, China; 3 Department of Hematology, Institute of Hematology, the First Affiliated Hospital, College of Medicine, Zhejiang University, Hangzhou, China; 4 Jiangsu Institute of Hematology, The First Affiliated Hospital of Soochow University, Key Laboratory of Thrombosis and Hemostasis, Ministry of Health, Suzhou, 215006, China; University of Kentucky, UNITED STATES

## Abstract

Previous studies in Chinese hamster ovary cells showed that truncational mutations of β3 at sites of F^754^ and Y^759^ mimicking calpain cleavage regulate integrin signaling. The roles of the sequence from F^754^ to C-terminus and the conservative N^756^ITY^759^ motif in platelet function have yet to be elaborated. Mice expressing β3 with F^754^ and Y^759^ truncations, or NITY deletion (β3-ΔTNITYRGT, β3-ΔRGT, or β3-ΔNITY) were established through transplanting the homozygous β3-deficient mouse bone marrow cells infected by the GFP tagged MSCV MigR1 retroviral vector encoding different β3 mutants into lethally radiated wild-type mice. The platelets were harvested for soluble fibrinogen binding and platelet spreading on immobilized fibrinogen. Platelet adhesion on fibrinogen- and collagen-coated surface under flow was also tested to assess the ability of the platelets to resist hydrodynamic drag forces. Data showed a drastic inhibition of the β3-ΔTNITYRGT platelets to bind soluble fibrinogen and spread on immobilized fibrinogen in contrast to a partially impaired fibrinogen binding and an almost unaffected spreading exhibited in the β3-ΔNITY platelets. Behaviors of the β3-ΔRGT platelets were consistent with the previous observations in the β3-ΔRGT knock-in platelets. The adhesion impairment of platelets with the β3 mutants under flow was in different orders of magnitude shown as: β3-ΔTNITYRGT>β3-ΔRGT>β3-ΔNITY to fibrinogen-coated surface, and β3-ΔTNITYRGT>β3-ΔNITY>β3-ΔRGT to collagen-coated surface. To evaluate the interaction of the β3 mutants with signaling molecules, GST pull-down and immunofluorescent assays were performed. Results showed that β3-ΔRGT interacted with kindlin but not c-Src, β3-ΔNITY interacted with c-Src but not kindlin, while β3-ΔTNITYRGT did not interact with both proteins. This study provided evidence in platelets at both static and flow conditions that the calpain cleavage-related sequences of integrin β3, i.e. T^755^NITYRGT^762^, R^760^GT^762^, and N^756^ITY^759^ participate in bidirectional, outside-in, and inside-out signaling, respectively and the association of c-Src or kindlin with β3 integrin may regulate these processes.

## Introduction

The role of platelets on cardio- and cerebro- vascular thrombotic diseases has been well established [1] and integrin αIIbβ3 is the most abundant membrane receptor in platelet serving as the last common pathway of platelet aggregation initiated by various agonists [2]. Allosteric changes of the αIIbβ3 integrin ectodomain regulated by agonist-induced intracellular signals, termed as inside-out signaling/activation, enable the platelets to bind fibrinogen with high affinity [3]. Once binding fibrinogen, αIIbβ3 integrin transduces signals in an outside-in direction, that mediate spreading and stable adhesion of platelets [4]. In contrast to the integrin αIIb subunit, the β3 subunit plays key roles in interacting with cytoplasmic proteins during signal transduction [5–7]. For instance, the membrane-proximal NPxY motif and the distal NxxY motif in β3 cytoplasmic tail, respectively binding talin [2,5] and kindlin (the latter is regarded as a coactivator for the former) [8,9], are supposed to regulate inside-out signaling (activation) of αIIbβ3 integrin, and C-terminal Arg-Gly-Thr (RGT) sequence, binding nonreceptor tyrosine kinase c-Src, is critical for outside-in signaling [6,10].

Once platelets bind fibrinogen via their receptor αIIbβ3 and aggregate subsequently, the calpain, a cystein protease, is activated [11]. Then, activated calpain cleaves the integrin β3 cytoplasmic tail progressively from C-terminus [10]. The cleavage of integrin β3 mediated by calpain was shown to suppress cell spreading, a typical event for outside-in signaling, in transfected Chinese hamster ovary (CHO) cell model [12] and the platelets from calpain-1 null mice showed enhanced spreading on collagen and fibrinogen-coated surfaces [13]. Fan et al. [14] reported that PIRB negatively regulated integrin αIIbβ3-mediated outside-in signaling, probably via promoting the calpain-cleavage of β3 Y^759^. Previous studies with CHO cell model showed that the truncational mutations mimicking calpain cleavage at β3 cytoplasmic domain at sites of F^754^ and Y^759^ differentially regulated the integrin bidirectional and outside-in signaling [10,15]. However, despite that αIIbβ3-expressing CHO cells have been widely applied as a model system that recapitulates the features of integrin signaling in platelets, data from this cell model need to be verified in real platelets.

In mouse platelets, it has been reported that the truncational mutation mimicking the calpain cleavage at the C-terminal 759 site (ΔRGT) resulted in a down-regulated outside-in signaling owing to the dissociation of c-Src [16]. Nevertheless, the effect of the calpain cleavage at F^754^ (ΔTNITYRGT) on integrin signaling in platelets is still unknown. Moreover, there is a genetically conserved NxxY motif between the F^754^ and Y^759^ cleavage sties, the NITY motif, which was shown to mediate inside-out signaling by interacting with kindlin [8,9,17,18]. Although the β3 membrane-distal NxxY motif from different species, including human, mouse, chicken, nematode, fruitfly, and zebrafish *etc*., exhibits a highly conserved sequence [19], the NxxY motif in different human β integrins displays a slight fluctuation in Y (Y for β1, β3, β5, and β6, but F for β2 and β7) [20,21]. Previous studies focused on point mutational analyses on Y [22], while there is a paucity of information about the deletion mutation of the entire NxxY motif. The strategy of complete deletion of motif, different from point mutation, has been used in cell function research [23]. In this study, we generated a whole-motif deletion mutation of β3 NITY to evaluate its roles in integrin signaling in platelets.

It is difficult to express recombinant proteins in short-life, anuclear platelets or in their megakaryocytic precursors [22]. Knock-in or knock-out β3 mutant mice generated through gene targeting of fertilized ova or embryonic stem cells are ideal models for the investigation of integrin β3 signal transduction [16,24], but this strategy is a time-consuming and low-throughput method, especially when multiple β3 mutants need to be studied. Establishing the mouse models with mutated β3 through transplanting hematopoietic stem cells (HSCs) or fetal liver cells which were infected by retroviral vectors encoding target genes into lethally radiated wild-type mice was reported as a high-throughput approach [22]. In current study we used this strategy [22] to generate mice whose platelets express different calpain cleavage-related β3 mutants.

Thrombus formation *in vivo* is affected by hemodynamic shear stress which can activate platelets on one hand, but also can wash or “tear” them off from adherent surface on the other hand [25]. Integrin αIIbβ3 signaling is required to resist this washing or “tearing” by shear stress [26–28]. Previous studies have established the importance of αIIbβ3 signaling in platelet function under static condition by using the β3-expressed platelets, information of these function under flow condition is however still largely lacking because of the technical difficulties. New microfluidic devices [29,30] with dimensions of micrometers provide higher shear rate within reasonable whole blood volume (0.1-1ml) requirement, making the small animal studies possible.

In this study, we generated mice whose platelets express the calpain cleavage-related β3 mutants (β3-ΔRGT, β3-ΔTNITYRGT, and β3-ΔNITY). Using these model platelets, we elucidated the role of specific β3 cytoplasmic sequences in regulating αIIbβ3 signal transduction in platelets under static and flow conditions. The mechanisms at a protein/protein interaction level regarding calpain cleavage-related β3 mutants with signaling molecules were also explored by GST prokaryotic expression system and the 293T cell model.

## Material and Methods

### Animals

The integrin β3-deficient homozygous mouse (β3^-/-^ mouse) on a C57BL/6 genetic background was generated as previous description [31] and was a generous gift from J. Liu (Shanghai Jiao Tong University School of Medicine, Shanghai, China). Wild-type C57BL/6 female recipient mice were purchased from SLRC Laboratory animal center (Shanghai, China). All animals were housed in groups (5 mice per cage) under a 12-h light/dark cycle (lights on at 08:00) at 23°Cin a specific pathogen-free environment and had ad libitum access to autoclaved food and water. The autoclaved cages were changed each week. Routine sanitation and environmental controls, including the temperature, humidity, ventilation, illumination and light schedule, and noise abatement, were performed by the animal care staff according to the related standards. If the mice suffering, they were gently removed to a new cage and were monitored more frequently. In addition, the anesthetics and analgesics could be used to alleviate the suffering during the experimental procedure.

### Ethics Statement

The animal study protocol was reviewed and approved by the Shanghai Jiao Tong University School of Medicine Institutional Animal Care & Use Committee in accordance with the guidelines of Shanghai Administration Rule of Laboratory Animal. The Protocol Registry Number was B-2015-010. All efforts were made to minimize suffering of mice.

Human platelets were obtained by collecting whole blood from healthy volunteers with informed consent. The protocol of collecting volunteers’ blood was approved by Ruijin Hospital Ethics Committee of Shanghai Jiao Tong University School of Medicine. The Protocol Registry number was 2014-2-20.

### Reagents and materials

PE-conjugated hamster anti-mouse integrin β3 (CD61) monoclonal antibody was purchased from BD pharmingen (Franklin Lakes, NJ). Alexa-Fluor 647 conjugated human fibrinogen was purchased from Molecular Probes. PE-conjugated anti-human β3 monoclonal antibody (CD61) for flow cytometry was purchased from eBioscience, Inc. (San Diego, CA). And the mouse anti-human β3 (SZ21) and anti-human αIIb (SZ22) monoclonal antibody for western blot and immunofluorescent assay were gifts from C. Ruan (Jiangsu Institute of Hematology, The First Affiliated Hospital of Soochow University) The rabbit antibodies against the β3 C-terminal TYRGT sequence, Ab762, or antibodies recognizing the calpain cleavage-generated C-terminus at each of the calpain cleavage sites, Ab759 and Ab754, were raised in our laboratory [10,15]. Goat anti-GST polyclonal antibody was purchased from GE Healthcare Life Sciences (Mississauga, Canada). Mouse anti-talin monoclonal antibody, rabbit anti-kindlin-3 antibody and rabbit anti-kindlin-2 antibody were purchased from Sigma-Aldrich (St Louis, MO). The kindlin family consists of three members in vertebrates, kindlin-1, kindlin-2 and kindlin-3. kindlin-1 and kindlin-2 are widely expressed, kindlin-3 is preferentially expressed in hematopoietic cells, mainly in megakaryocytes and platelets [9,17]. Rabbit anti-Src monoclonal antibody was purchased from Cell Signaling Technology, Inc. (Boston, MA). Alexa Fluor 594 conjugated AffiniPure goat anti-rabbit IgG secondary antibody was purchased from Jackson ImmunoResearch Laboratories, Inc. (West Grove, PA) and Alexa Fluor 488 conjugated goat anti-mouse IgG secondary antibody was from ThermoFisher Scientific (Waltham, MA). Actin staining was performed using tetramethyl rhodamine isothiocynate (TRITC)-conjugated phalloidin purchased from Sigma-Aldrich (St Louis, MO). Quickchange Lightning Site-Directed Mutagenesis Kit was purchased from Agilent Technologies (Santa Clara, CA). Calcium phosphate cell transfection kit was a product of Beyotime Institute of Biotechnology (Jiangsu, China). Recombinant mouse stem cell factor (MSCF), IL-3, and IL-6 were purchased from R&D (Minneapolis, MN). Peptides RGDS (Arg-Gly-Asp-Ser) and protease activated receptors 4 (PAR4)-thrombin receptor activating peptide (Ala-Tyr-Pro-Gly-Lys-Phe [AYPGKF]) were synthesized at GL Biochem (Shanghai, China). Collagen type I and adenosine diphosphate (ADP) were purchased from Chrono-log Corporation (Havertown, PA). Purified human fibrinogen was purchased from Enzyme Research Laboratories (South Bend, IN). All other biochemical reagents were obtained from Sigma-Aldrich (St Louis, MO).

### Retrovirus construction

The cDNA of murine integrin β3 was cloned by reverse transcription polymerase chain reaction (RT-PCR) from C57BL/6 spleen total RNA. The mutants of the cytoplasmic tail of integrin β3 were generated using PCR (β3-ΔTNITYRGT and β3-ΔRGT) or Quickchange Lightning Site-Directed Mutagenesis Kit (β3-ΔNITY). Primers used were as follows: β3-ΔRGT and β3-ΔTNITYRGT common forward: GGGTCCTGATATCCTG, β3-ΔRGT reverse: GAATTCTTAGTAGGTGATATTGGTG, β3-ΔTNITYRGT reverse: GAATTCTTAGAAGGTGGAGGTGGCC. β3 wild-type, β3-ΔRGT and β3-ΔTNITYRGT cDNA were subcloned into the MSCV MigR1 retroviral plasmid with an IRES-GFP inserted prior to the polyadenylation signal as previously described [31]. β3-ΔNITY retrovirus were generated from MSCV MigR1 retrovirus with β3 wild-type using Quickchange Lightning Site-Directed Mutagenesis Kit. Primers used were as follows: β3-ΔNITY forward: GGCCACCTCCACCTTCACCCGGGGGACTTAAGAATTCC, β3-ΔNITY reverse: GGAATTCTTAAGTCCCCCGGGTGAAGGTGGAGGTGGCC.

### Genotyping of β3^-/-^ mice

The β3^-/-^ mice were identified by PCR according to previous publication [31].

#### Bone marrow transplantation

Bone marrow mononuclear cells (MNCs) were harvested from 6-week-old male β3^-/-^ mice and cultured in DMEM with a supplement of murine stem cell factor (MSCF), IL-3, and IL-6. Then the bone marrow MNCs were transfected twice with retrovirus encoding different mutant β3 cytoplasmic tails. 1×10^6^ bone marrow MNCs were transplanted by caudal vein injection (200 μl per mouse) into every recipient female wild-type mouse (8 to 10 weeks old) conditioned with a lethal dose of 850 cGy α-ray total body irradiation. Four to eight weeks after transplantation, the platelets of mice were tested for the β3 and GFP expression by collecting blood every week. About one in ten transplanted mice died prior to the experimental endpoint because of the failure of bone marrow substitution. The mice were monitored and evaluated twice a day during the experimental procedure. Mice were sacrificed by CO2 inhalation when they showed the clinical signs, such as the reluctance to move when undisturbed, a hunched still posture, back arching, twitching muscular spasms and dyspnea, prior to the experimental endpoint.

### β3 and GFP expression using flow cytometry

Ten microliters of whole blood containing the anticoagulant sodium citrate was collected from β3^+/+^, β3^+/-^ and β3^-/-^ mice, as well as transplanted mice by cutting tail. The whole blood diluted with PBS was incubated with PE-conjugated hamster anti-mouse integrin β3 (CD61) at 1:50 at room temperature for 30 minutes. The β3 expression of platelets was measured with flow cytometry. For the transplanted mice the GFP expression was tested simultaneously and the fluorescence intensity of β3 expression in GFP-positive platelets gated was calculated.

### Blood collection

Six to eight weeks after transplantation, the mice were anesthetized with 2% pentobarbital and 900 μl-1,000 μl whole blood was collected by cardiac puncture with an injector containing 3.8% sodium citrate. After collecting blood, the anesthetized mice were euthanatized by using CO_2_ inhalation. All efforts were made to minimize suffering of mice. For the whole blood, 500 μl was put aside for flow assay and the remaining part was used to prepare platelet-rich plasma (PRP) by a centrifugation at 200 g for 5 minutes. Then PRP was acidified by adding 1/4 volume of acid-citrate-dextrose (ACD; 38 mM citric acid, 75 mM trisodium citrate,136 mM glucose), and centrifuged at 400 g for 5 minutes. The platelet pellets were washed twice with CGS buffer (120 mM NaCl, 13 mM trisodium citrate, 30 mM glucose, pH 6.5) at 300 g for 5 minutes and were finally resuspended with HEPES-Tyrode’s buffer (137 mM NaCl, 2 mM KCl, 12 mM NaHCO_3_, 0.3 mM NaH_2_PO_4_, 1 mM CaCl_2_, 1 mM MgCl_2_, 5.5 mM glucose, 5 mM HEPES, 0.1% BSA, pH 7.4). The platelet suspensions were rested at room temperature for one hour prior to being used in experiments. β3^-/-^, β3^+/-^ and β3^+/+^ mice served as control.

### Fibrinogen binding assay

Soluble fibrinogen binding assay was performed as previously described [22,32]. Washed platelets were resuspended at in HEPES-Tyrode’s buffer 2×10^6^/ml and then were stimulated with 0.5 mM Mn^2+^ (in MnCl_2_ solution), 50 μM ADP accompanied with 5 μM epinephrine (Epi) (ADP/Epi), 0.5 mM PAR4 peptide, or no agonists for 30 minutes at 37°C. After stimulation, the platelets were incubated with 100 μg/ml of Alex-Fluor 647-conjugated fibrinogen for 30 minutes at 37°C in dark. The reaction was stopped by fixation with 4% formaldehyde for 15 minutes at room temperature. Then the platelets were washed with PBS by centrifugation at 800 g for 5 minutes. Fibrinogen binding of total platelets or GFP positive platelets gated was tested with an EPICS XL flow cytometer (Beckman Coulter) and the data were analyzed with FlowJo software. Specific fibrinogen binding was calculated by total binding minus nonspecific binding in the absence of any agonists. Samples treated with 2 mM RGDS served as antagonists.

### Spreading assay

Platelet spreading assays were performed as described in the literature [33]. The Lab-Tek chamber slides (Nalge Nunc International) were precoated with 20 μg/mL of human fibrinogen overnight at 4°C, and blocked with 2% BSA for three hours at room temperature after washing with PBS. Washed platelets, resuspended at a final concentration of 2×10^6^/mL in HEPES-Tyrode’s buffer, were allowed to adhere and spread on fibrinogen-coated slides at 37°C for 120 minutes in the presence of 100 μM ADP, 0.5 mM PAR4 peptide, or no agonists. After washing three times with PBS, the attached platelets were fixed with 4% paraformaldehyde (PFA) for 15 minutes at 4°C, permeabilized, and stained with TRIFC-labeled phalloidin as previously described [34]. Finally, the coverslips were mounted on microscopy glass slides using Mowiol/DABCO. Single fluorescent image was collected on a conventional fluorescence microscope (Leica Leitz) with a 60× oil immersion objective and a Leica DC 300F camera using the Leica IM1000 1.20 software. The images of GFP and actin staining were overlaid using Photoshop software, and only platelets with both GFP and actin staining were analyzed. The calculation of the surface areas of 50–74 GFP-positive platelets from at least three transplanted mice for each mutant was done using NIH Image J software (http://rsbweb.nih.gov/ij/) [16,22,34,35].

### Adhesion assay under flow

*Ex vivo* flow-based platelet adhesion assay was performed essentially as described [36,37]. Briefly, microfluidic channels with the cross section of 250 μm in width×75 μm in height (Bioflux 200 from Labtech, Fluxion Biosciences Inc.) were coated with 100 μg/ml fibrinogen or 20 μg/ml collagen at 4°C overnight followed by blocking with 2% BSA. Because of the disparity in the ratio of GFP-positive platelets from different transplanted mouse, the whole blood of β3^-/-^ mice was added into the whole blood of transplanted mice to calibrate the number of GFP-positive platelets according to the GFP-positive ratios and whole platelet counts. The modulated whole blood was perfused through microcapillary tubes at a wall shear rate of 125 s^-1^ for 12 minutes for adhesion to fibrinogen-coated surface or at 1,500 s^-1^ for 5 minutes for adhesion and aggregation to collagen-coated surface. GFP-positive platelets were monitored in real time (acquisition rate: 10 frame every 1 second) under flow using an inverted fluorescent microscope and CCD camera (Nikon eclipse Ti-s). The data were analyzed using the Bioflux 200 software. The number of adherent GFP-positive platelets on fibrinogen or coverage area of aggregated GFP-positive platelets on collagen from 10–15 randomly selected visual fields from different transplanted mice was analyzed. The ratio of the number or coverage area of adherent GFP-positive platelets to that of total GFP-positive platelets was defined as adhesion ratio on fibrinogen or collagen.

### Plasmid constructs for prokaryotic expression and purification

The cDNA fragments encoding the wild-type β3 cytoplasmic tail (residues 716–761) were generated from the corresponding full-length constructs [10] using primers introducing BamH1 and XhoⅠrestriction sites at the 5’- and 3’-ends, respectively and cloned into the pGEX-6P1 vector (GE Healthcare) downstream of the GST sequence (GST-β3). The mutants of the cytoplasmic tail of β3 were generated using Quickchange Lightning Site-Directed Mutagenesis Kit (GST-β3-ΔRGT, GST-β3-ΔTNITYRGT, and GST-β3-ΔNITY). Mutagenic primers were designed using web-based QuikChange Primer Design Program available online at www.agilent.com/genomics/qcpd. Primers used were as follows: β3-ΔRGT forward: TTCACCAATATCACGTACTAAGGCACTTAACTCGAG, β3-ΔRGT reverse: CTCGAGTTAAGTGCCTTAGTACGTGATATTGGTGAA; β3-ΔTNITYRGT forward: GCCCCGGTACGTGATATTGGTTTAGGTAGACGTGGCCTCTTTATA, reverse: TATAAAGAGGCCACGTCTACCTAAACCAATATCACGTACCGGGGC; β3-ΔNITY forward: CGAGTTAAGTGCCCCGGGTGAAGGTAGACGTG, β3-ΔNITY reverse: CACGTCTACCTTCACCCGGGGCACT`TAACTCG. All constructs were verified by DNA sequencing.

GST alone, GST-β3 wild-type or GST-β3 mutated fusion proteins (GST, GST-β3, GST-β3-ΔRGT, GST-β3-ΔTNITYRGT, and GST-β3-ΔNITY) were expressed in Escherichia coli BL21 (DE3) and purified from bacterial lysates by batch elution from glutathione-Sepharose (GE Healthcare Life Sciences). These purified GST fusion proteins were identified by western blot with antibodies specifically recognizing β3 amino acid residues after deletion of TNITYRGT and RGT (Ab 754, and Ab 759) and with an antibody recognizing the COOH terminus of (Ab 762).

### GST pull-down assays

30 μg of purified GST fusion proteins coupled to glutathione-Sepharose 4B beads (GE Healthcare Life Sciences) were incubated overnight at 4°C with aliquots of human platelet lysates in lysis buffer (0.5% NP-40, 50 mM HEPES, pH 7.7, 150 mM NaCl, 0.1 mM EDTA, 1 mM PMSF and the protease inhibitor cocktail). Complexes were washed and subjected to western blot analysis using specific anti-talin, kindlin-3, and c-Src antibodies.

### Expression of integrin αIIb/wild-type or mutant β3 in 293T cells and immunofluorescencet assays

The plasmid pcDNA3.1(-)/β3 wild-type and pcDNA3.1(-)/αIIb were gifts from N. Kiefer (Sino-French Research Center for Life Sciences and Genomics, Ruijin Hospital)[19]. The plasmids with mutant β3 (pcDNA3.1(-)/β3-ΔRGT, pcDNA3.1(-)/β3-ΔTNITYRGT, and pcDNA3.1(-)/β3-ΔNITY) were generated from pcDNA3.1(-)/β3 using Quickchange Lightning Site-Directed Mutagenesis Kit. Each pcDNA3.1(-)/β3 wild-type or β3 mutants was co-transfected together with pcDNA3.1(-)/αIIb into 293T cells (Human embryonic kidney cells) using a calcium phosphate cell transfection kit. The cells were selected using a G418 selection medium and analyzed by flow cytometry using a PE-conjugated anti-human β3 monoclonal antibody along with a mouse anti-human αIIb antibody, SZ22, an Alexa Fluor 488 conjugated goat anti-mouse IgG. These β3-positive cell populations were enriched by cell sorting, and then subcloned by limiting dilution. The levels of β3 and αIIb expression were measured by flow cytometry and western blot.

Stably transfected cells suspended in HEPES-Tyrode’s buffer were added to fibrinogen-coated slides and incubated at 37°C for 120 min. After washing,the cells were fixed with 4% paraformaldehyde,permeabilized with Labeling buffer (0.5% Triton-100, 0.5% BSA, 1×PBS),and incubated with the mouse anti-human β3 antibody, SZ21, and the rabbit anti-kindlin-2 antibodies or rabbit anti-Src antibody. After washing, the cells were stained with Alexa Fluor 488 conjugated goat anti-mouse IgG and Alexa Fluor 594 conjugated goat anti-rabbit IgG. Data were collected using Leica laser confocal microscope (Leica TCS SP8; Leica Microsystems, Wetzlar, Germany).

### Statistical analysis

The SPSS 18 statistical software program was employed for the statistical analysis. Statistical significance between different groups was carried out using one-way analysis of variance (ANOVA). Quantitative data were expressed as means ± SEM. P values less than 0.05 or 0.01 were considered to be statistically significant or clearly significant.

## Results

### Retroviral expression of wild-type β3 restores inside-out and outside-in signaling responses in β3-deficient platelets

**Fig 1:**
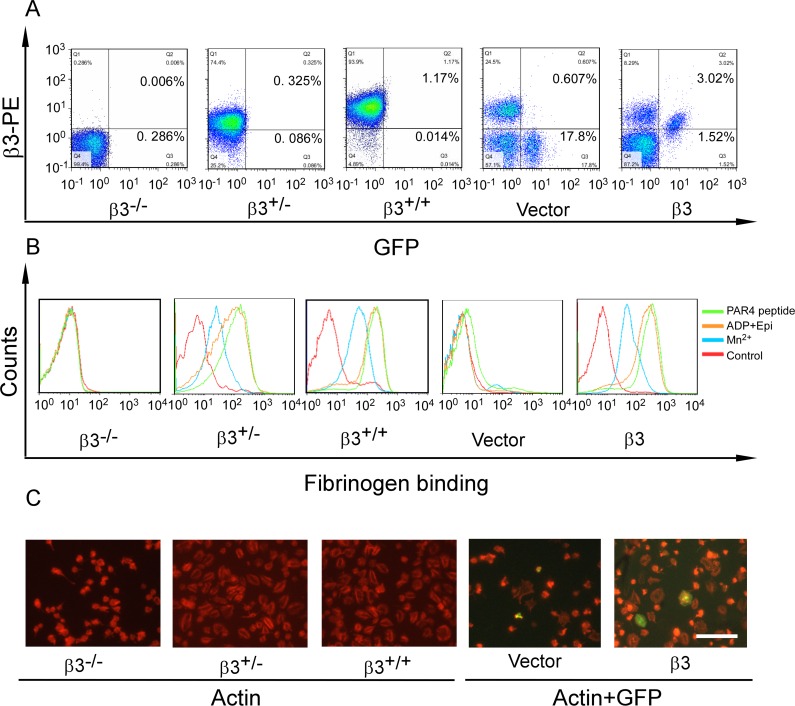
Retroviral β3 expression enables bidirectional signaling. (A) The expression of β3 and GFP in the platelets of vector and β3 transplanted mice, as well as β3 deficient homozygote (β3^-/-^), heterozygote (β3^+/-^), and wild-type (β3^+/+^) mice. (B) Alexa-Fluor 647-conjugated fibrinogen binding was measured in β3^-/-^, β3^+/-^, β3^+/+^ platelets and in GFP-positive platelets gated by flow cytometry from transplanted animals upon Mn^2+^ (blue lines), ADP/Epi (orange lines) and PAR4 peptide (green lines) stimulation or no agonist (red lines for control). The detailed scatter diagram was shown in S1 Fig (C) Spreading of β3^-/-^, β3^+/-^, β3^+/+^ platelets and GFP-positive platelets of transplanted mice (The GFP and β3 expressions in the platelets are shown in A.) on immobilized fibrinogen in presence of PAR4 peptide. Platelets were visualized using tetramethyl rhodamine isothiocynate-conjugated phalloidin. The scale bar represents 40 μm.

**Fig 2:**
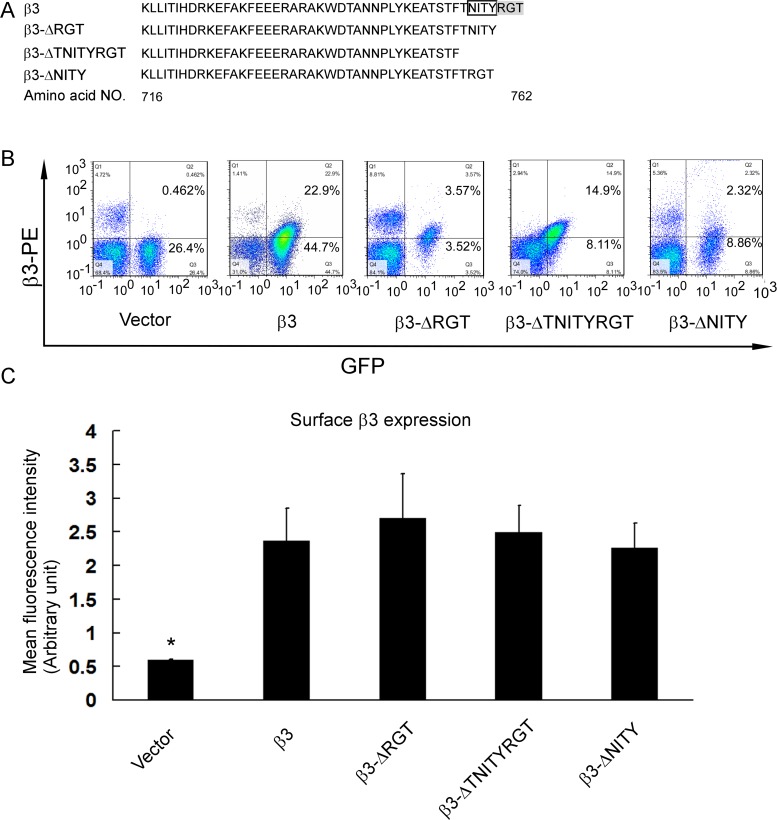
β3 expression of GFP-positive platelets in transfected platelets. (A) The amino acid sequences of the cytoplasmic tail of wild-type and mutated β3. DNA fragment was also sequenced (S2 Fig). (B) Integrin β3 and GFP expression in platelets of representative transplanted mice. (C) Statistical histogram of mean fluorescence intensity (mean±SEM) of β3 expression in GFP-positive platelets from at least three individual animals for each type of mutants. *P<0.05, compared to the wild-type β3 expressing platelets.

By transplanting β3^-/-^ mouse HSCs infected by retroviral vectors encoding different β3 sequences into lethal radiated wild-type mice, we successfully established mice producing platelets that express wild-type and mutant β3 (β3, β3-ΔRGT, β3-ΔTNITYRGT, β3-ΔNITY, and vector) in a β3 deficient background (Figs 1 and 2).

To test whether the platelets transfected with wild-type β3 are capable of undergoing bidirectional signaling, soluble fibrinogen binding and spreading on fibrinogen-coated surface, typical events of inside-out signaling and outside-in signaling respectively, were assayed. The platelets co-expressing β3 and GFP showed an increased fibrinogen binding after stimulated by agonists such as Mn^2+^, ADP/Epi, and PAR4 peptide, while platelets with a control vector expressing only GFP did not (Fig 1B and S1 Fig). Likewise, β3 and GFP co-expressing platelets spread on the fibrinogen-coated surface after 2 hours that was enhanced in presence of PAR4 peptide, while the platelets with only GFP-expression did not, similar to the β3^-/-^ platelets (Fig 1C). These data indicated that retroviral complementation of β3 restores inside-out as well as outside-in signal transduction enabling this model to be applied in studying integrin signaling and platelet function.

### Expression of β3 with or without cytoplasmic tail mutants in platelets of transfected mice

The amino acid sequences and DNA sequences of wild-type and different β3 mutants were shown (Fig 2A and S2 Fig). Analysis of whole blood of the transplanted mice revealed mild anemia and normal platelet count, in comparison with the wild-type mice (S1 Table). The blood counts of β3^+/+^, β3^+/-^ and β3^-/-^ mice were lower than previous report [31], probably resulting from the difference of utilized methods (S1 Table). Flow cytometry showed that the GFP expression ranged from 1.6% to 67.6% (Figs 1A and 2B), which was consistent with the literature [22]. The platelets expressing β3 in the absence of GFP might be from wild-type recipient HSCs that escaped from irradiation even though at lethal doses. The efficiency of reconstitution varied and there were differences in the amount of wild-type recipient platelets among the groups. The mice with more than 4.5% GFP-positive platelets were enrolled in this study. The GFP-positive platelets of the transplanted mice expressed β3 to a level similar to the β3^+/-^ mouse platelets (Fig 1B). No statistical difference of β3 expression was observed in the transfected platelets expressing wild-type and mutated β3 (β3-ΔRGT, β3-ΔTNITYRGT, and β3-ΔNITY) (at least 3 mice for each mutant) (Fig 2C). We also found that erythrocytes scarcely express GFP, albeit platelets and bone marrow MNCs did in successfully transplanted mice (S3 Fig), which facilitates the observation of the adhesion of GFP-positive platelets in whole blood to the fibrinogen- or collagen-coated surface under flow in the presence of a large number of erythrocytes. These GFP-negative erythrocytes might be derived from the recipient HSCs since the erythrocytes have a life-time longer than platelets and leucocytes.

### Soluble fibrinogen binding of platelets with different β3 mutants

To test the ability of the platelets with different β3 mutants to transmit inside-out signals, fibrinogen binding was measured in these platelets by gating GFP-positive platelets following stimulation by agonists including Mn^2+^, ADP/Epi, and PAR4 peptide. Given that β3-expression level exhibited slight disparities among the platelets of different transplanted mice, mean fluorescence intensity (MFI) for every agonist treatment was normalized to the MFI obtained with Mn^2+^ treatment, since Mn^2+^ can directly cause conformation change of integrin αIIbβ3 extracellular domain required for fibrinogen binding without a need of participation of the cytoplamic domains [16,38]. Fibrinogen binding stimulated by Mn^2+^ reflects the quantity of β3 in the platelet membrane [38], while that stimulated by ADP (binding to P_2_Y_12_, P_2_Y_1_ receptors [39])/Epi (binding to adrenergic receptors α_2A_ and sensitizing platelets to the action of ADP [40,41]), or PAR4 peptide (binding to PAR4 [39]) requires the mediation of inside-out signaling.

**Fig 3:**
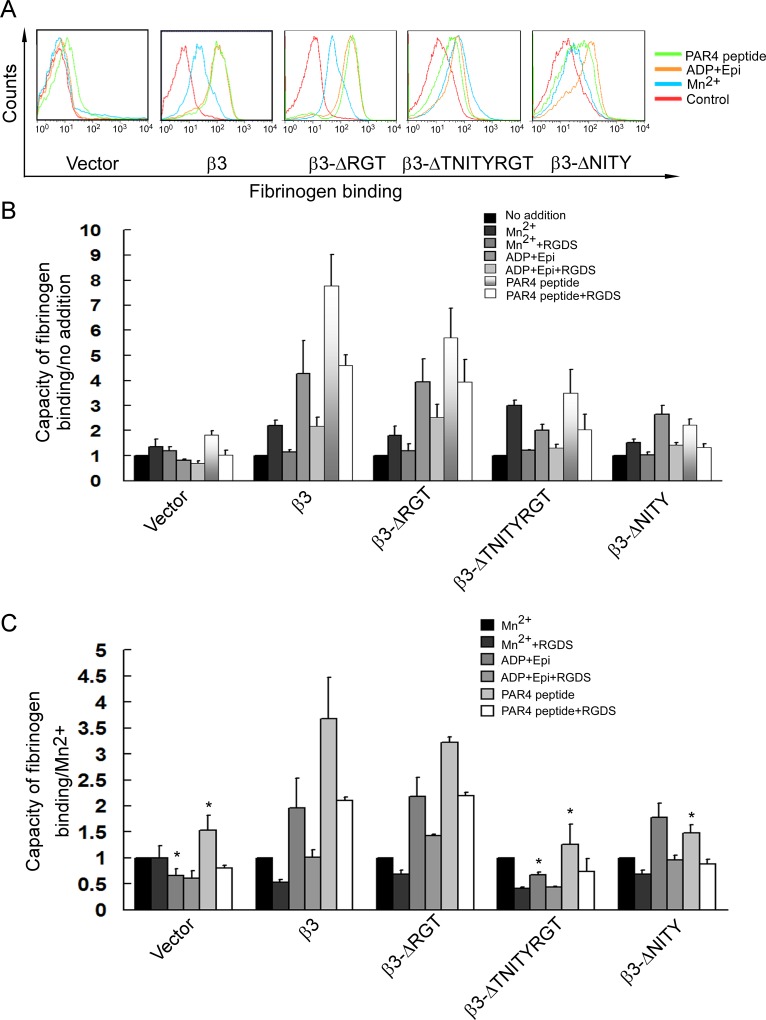
Loss of the β3 cytoplasmic NITY motif, rather than RGT motif, impairs inside-out αIIbβ3 signaling. Alexa-Fluor 647-conjugated fibrinogen binding was measured in GFP-positive platelets gated by flow cytometry from vector, β3, β3-ΔRGT, β3-ΔTNITYRGT and β3-ΔNITY transplanted mice upon Mn^2+^ (blue lines), ADP/Epi (orange lines) and PAR4 peptide (green lines) stimulation, or no agonist (red lines for control). (A) The representative images of fibrinogen binding. (B) The mean fluorescent intensity (MFI) of fibrinogen binding in the present of agonists (Mn^2+^, ADP/Epi, or PAR4 peptide) or antagonists (RGDS) was calculated based on the basal level fibrinogen binding platelets without treatment by agonists or antagonists. (C) The mean fluorescent intensity of fibrinogen binding with agonists (ADP/Epi, or PAR4 peptide) or antagonists (RGDS) was calibrated with that stimulated by Mn^2+^. Statistical chart is from at least three individual animals so performed (mean±SEM). *P<0.05, compared to the wild-type β3 transfected platelets.

Representative graphs of fibrinogen binding of GFP-positive platelets with different β3 mutants in the presence or absence of agonists was shown (Fig 3A) and statistical chart of normalized MFI from a certain number of mice was also obtained (Fig 3B and 3C). A complete defect in fibrinogen binding was observed in β3-ΔTNITYRGT platelets, similar to vector-transfeted platelets. In contrast, β3-ΔRGT platelets completely preserved the capability to bind fibrinogen, similar to β3 wild-type platelets. Meanwhile, fibrinogen binding was partially impaired in β3-ΔNITY platelets. Fibrinogen binding stimulated by ADP/Epi or PAR4 peptide could be most inhibited by RGDS peptide at a scale similar to those published elsewhere [42,43] indicating the specificity of the binding (Fig 3B and 3C). Fibrinogen binding slightly increased in vector-transfected platelets uniquely after PAR4 peptide stimulation, while did not in β3^-/-^ platelets (Figs 1B and 3). This unexpected phenomenon needs further research.

### Spreading on immobilized fibrinogen of platelets with different β3 mutants

**Fig 4:**
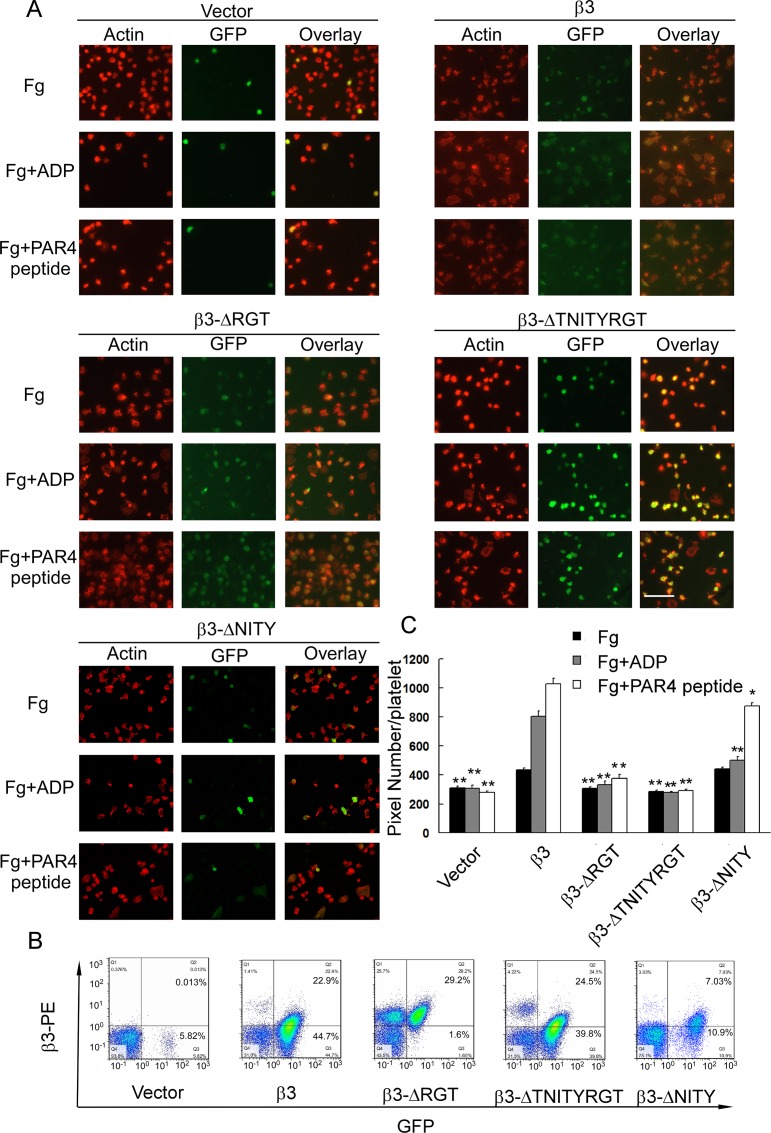
Outside-in αIIbβ3 signaling requires the β3 C-terminal RGT motif, but not NITY motif. Platelet of transplanted mice spreading on immobilized fibrinogen only (Fg), immobilized fibrinogen accompanied with ADP (Fg+ADP), or PAR4 peptide (Fg+PAR4 peptide). (A) The representative images of actin staining and GFP expression under fluorescence microscopy. The scale bar represents 40 μm. The β3 and GFP expressions of the representative transfected platelets are shown in Panel B. The green fluorescence of β3 wild-type and β3-ΔRGT transfected platelets looks dim (A), because the mean green fluorescence intensities of them are relatively low in comparison to other kinds of transfected platelets (B). Several GFP-negative platelets are observed to spread well in response to stimulation in β3-ΔRGT and β3-ΔTNITYRGT platelet groups, but not in the vector ones (A). That is because there is almost no recipient platelet left in vector transfected mice (B). The GFP-positive ratio of the wild-type β3 group (A) looks higher than that shown in flow cytometry (B), probably resulting from a loss of GFP^-^β3^-^ platelets by washing during the process of slide preparation. (C) The area occupied by GFP-positive adherent platelets was measured using the Image J program. The results are the mean ± SEM from 20–60 GFP-positive individual platelets of at least three animals analyzed for each type of mutants. *P<0.05 and **P<0.01, compared to the wild-type β3 transfected platelets.

We further observed the spreading of GFP-positive platelets with different β3 mutants on fibrinogen-coated surface, the typical event of outside-in signaling. We also added ADP or PAR4 peptide to stimulate the platelets according to the literature [16,38], in view of the fact that mouse platelets are small and spread poorly in the absence of agonists [38]. The β3-ΔRGT platelets with an intact potential to bind soluble fibrinogen (Fig 3) demonstrated a significant defect in platelet spreading, as compared with the wild-type β3, in the absence and presence of agonists (ADP or PAR4 peptide), just like vector-transfeted platelets (Fig 4) (P>0.05, when compared to vector transfected platelets). No matter whether the agonists existed or not, the β3-ΔTNITYRGT platelets exhibited a complete defect in platelet spreading, similar to vector transfected platelets (Fig 4) (P>0.05, when compared to vector transfected platelets). The β3-ΔNITY platelets displayed almost a normal spreading in the absence of agonists and a slight decrease in spreading in the presence of agonists (Fig 4) (P<0.05, when compared to vector transfected platelets; P<0.05, when compared to wild-type β3 transfected platelets).

### Adhesion of platelets with different β3 mutants under flow

**Fig 5:**
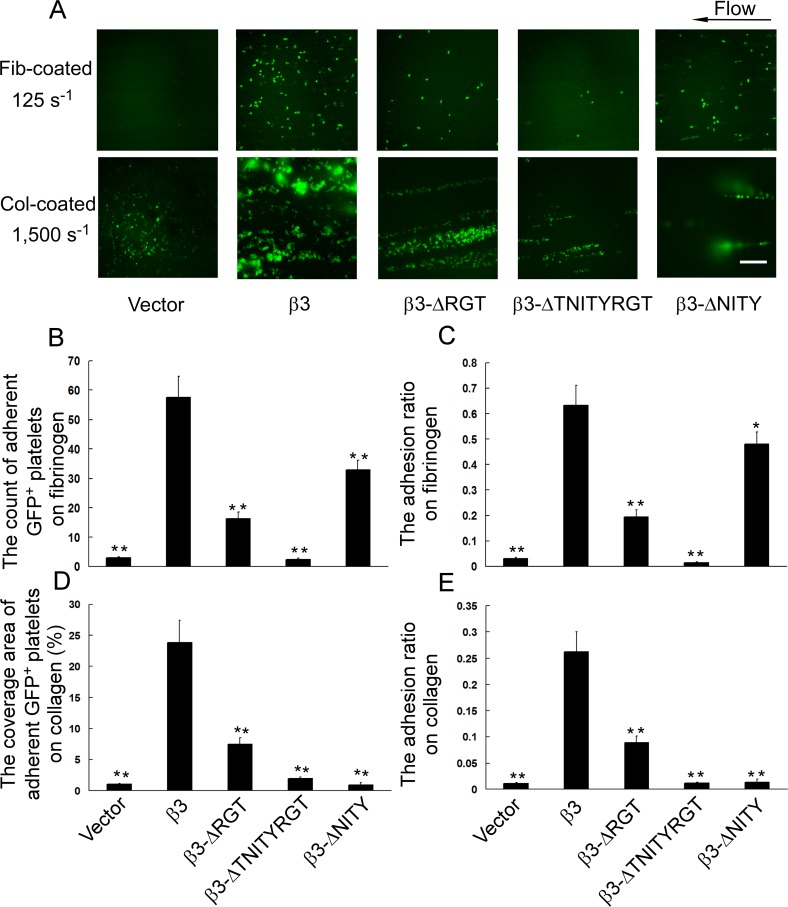
Effects of different β3 mutations on platelet function under flow. Whole blood from transplanted mice was perfused through the capillary tubes coated with fibrinogen or collagen at 125 s^-1^ for 12 minutes or 1,500 s^-1^ for 5 minutes, respectively. The adherent GFP-positive platelets were recorded in real-time under fluorescence microscopy. (A) The representative images of adherent GFP-positive platelets on fibrinogen or collagen-coated surface. The scale bar represents 40 μm. (B) The number of adherent GFP-positive platelets on fibrinogen. (C) The adhesion ratio on fibrinogen (relative value). (D) The coverage area of adherent and aggregated GFP-positive platelets on collagen. (E) The adhesion ratio on collagen (relative value). Results are the mean ± SEM from 10–15 randomly selected visual fields for each type of mutants. *P<0.05 compared to the wild-type β3 transfected platelets.

To further investigate the ability of platelets with different β3 sequences to resist the hydrodynamic drag forces, we observed the stability of the platelets with different β3 mutants to adhere to fibrinogen or collagen-coated surface under flow. At shear rate of 125 s^-1^, the number of adherent GFP-positive platelets to fibrinogen-coated surface showed a slight decrease for β3-ΔNITY, an obvious decrease for β3-ΔRGT, and a drastic decrease for β3-ΔTNITYRGT (Fig 5A and 5B) and so did the adhesion ratio (Fig 5C). At arteriolar shear rate of 1,500 s^-1^, the coverage area of adherent and aggregated GFP-positive platelets on collagen-coated surface showed a partial diminution for β3-ΔRGT, and a profound one for β3-ΔTNITYRGT and β3-ΔNITY (Fig 5A and 5D) and so did the adhesion ratio (Fig 5E).

### Interaction of different β3 mutants with signaling molecules

**Fig 6:**
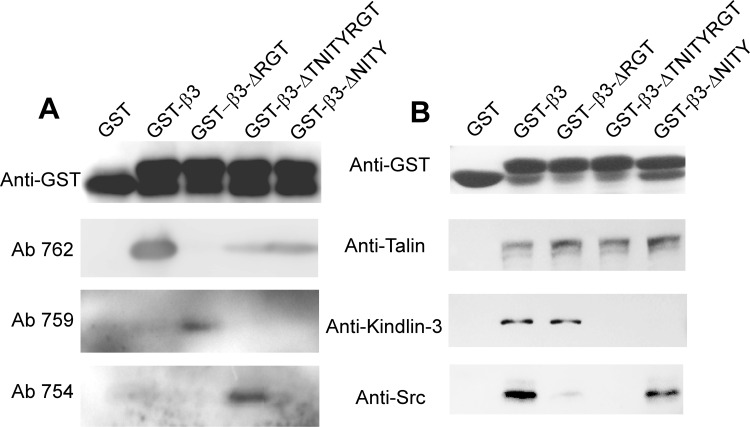
Interaction of different β3 (WT and mutants) with signaling molecules. (A) Expression of correct truncational mutants in each of the GST-β3 cytoplasmic tail fusion proteins was verified with antibodies specifically recognizing calpain cleaved forms of β3 (Ab 759 and Ab 754) and an antibody recognizing the COOH terminus of (Ab 762). (B) Glutathione-Sepharose 4B beads coated with GST-β3 cytoplasmic tail fusion proteins were incubated overnight with platelet lysates at 4°C. After washing the special antibodies were used to detect talin, kindlin-3, and c-Src binding. Anti-GST antibody was used to verify the loading of the β3 cytoplasmic tail fusion proteins.

To investigate the mechanism of different β3 mutants in regulating platelet function, we built the GST-tagged β3 cytoplasmic tail fusion proteins and tested their interaction with signaling molecules. GST fusion proteins with different β3 mutations were proved to be correct with antibodies specifically recognizing β3 amino acid residues after deletion of RGT and TNITYRGT (Ab 759 and Ab 754) and with an antibody recognizing the COOH terminus of (Ab 762) (Fig 6A). The pull-down assays showed that GST-β3-ΔRGT preserved the ability to bind talin and kindlin-3, but not c-Src, and GST-β3-ΔNITY can bind talin and c-Src, but not kindlin-3, while GST-β3-ΔTNITYRGT can only bind talin (Fig 6B).

**Fig 7:**
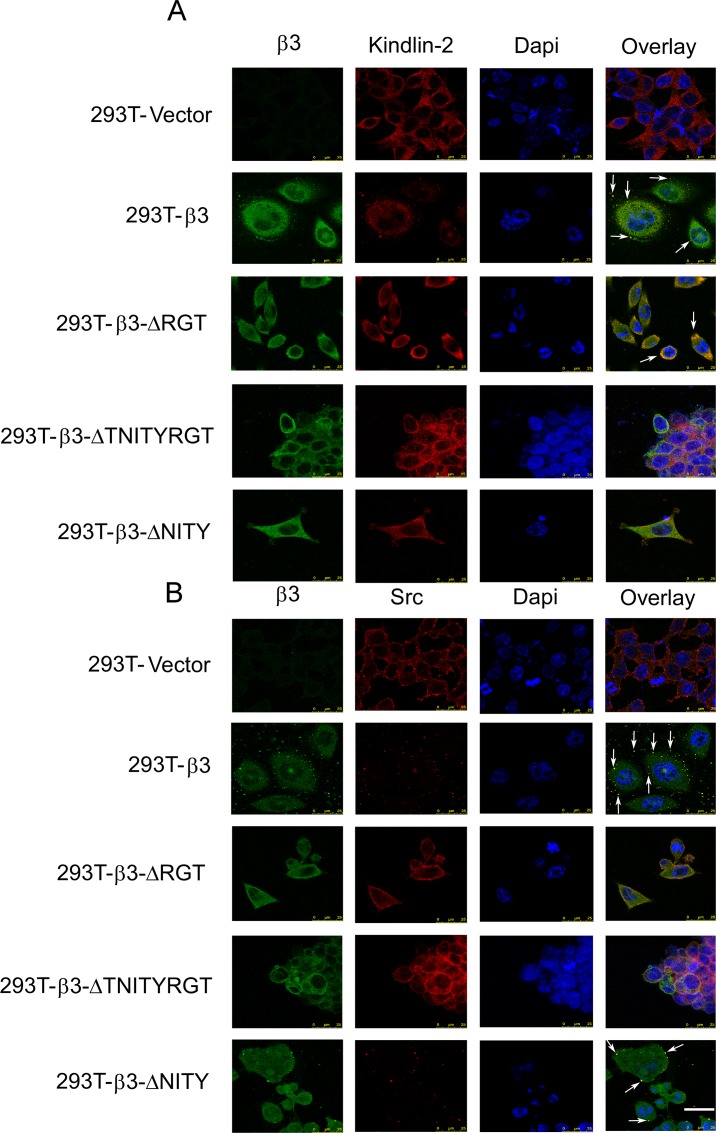
Co-localization of different β3 (WT and mutants) with signaling molecules. Stably transfected cells (As characterized in S6 Fig) were allowed to spread on fibrinogen-coated slides for 120 min, fixed, and permeabilized. (A) The slides were stained with a mouse anti-β3 monoclonal antibody, SZ21 (green), and rabbit anti-kindlin-2 antibodies (red), followed by fluorescence-labeled secondary antibodies, as well as Dapi (blue). (B) Methods applied were similar to Panel A, except the use of rabbit anti-Src antibodies (red). Data were collected with a Leica laser confocal microscope. The scale bar represents 25 μm.

Furthermore, we explored the intracellular interaction of different β3 mutations with kindlin and c-Src by immunofluorescent assay. Human 293T cells stably transfected with mutated β3 and wild-type αIIb (S6 Fig) were used to substitute the platelets from transplanted mice. In cells spreading on immobilized fibrinogen, β3 wild-type and β3-ΔNITY enabled the co-localization with c-Src, but β3-ΔRGT and β3-ΔTNITYRGT did not. In contrast, β3-ΔRGT, just like β3 wild-type, could co-localize with kindlin-2, while the β3-ΔTNITYRGT and β3-ΔNITY could not (Fig 7). The results from co-localization were quite consistent with those from pull-down.

## Discussion

Integrin αIIbβ3 mediates bidirectional signaling in which the β3 subunit is responsible for interacting with cytoplasmic signaling molecules in most cases. The amino acid mutations or motif deletions in β3 were shown to result in an influence on integrin signaling. For example, the diYF mice bearing substitutions at β3 Y^747^ and Y^759^ residues with phenylalanines exhibited a defect in αIIbβ3 outside-in signaling [24]. Mutually exclusive binding of talin and Gα_13_ to β3 membrane-proximal E^731^EE^733^ motif switches the direction of αIIbβ3 signaling and mutation of this motif causes a damaged spreading on immobilized fibrinogen [44,45]. Zou *et al*. [22] found that platelets with a mutation at β3 C-terminal mere last residue T^762^ displayed an impaired spreading. The β3 (ΔRGT) knock-in mice featured an impairment mainly on outside-in signaling [16]. When platelets are activated by agonists, the β3 cytoplasmic domain can be cleaved by calpain gradually from the C-terminus [10]. Truncational mutations mimicking calpain cleavage at the β3 cytoplasmic domain was previously shown in CHO cell model to differentially regulate the integrin bidirectional signaling [10]. In the present study, platelets bearing β3 mutants (β3-ΔRGT, β3-ΔTNITYRGT, and β3-ΔNITY) mimicking cleavage during platelet activation were assayed for signal transduction by testing their ability to bind soluble fibrinogen and spread on immobilized fibrinogen.

Soluble fibrinogen binding to αIIbβ3 depends on the transformation of αIIbβ3 ectodomain to higher affinity. Mn^2+^ can directly cause this transformation without a need of the participation of signal transduction. Mn^2+^-induced fibrinogen binding is generally used to evaluate the potential of αIIbβ3 to be activated in experiments applying recombinant β3 [16,38]. We also found that the MFI of fibrinogen binding under Mn^2+^ stimulation was closely related to the β3 expression (S4 Fig). On the other hand, intracellular signals are obligatory for the physical agonists such as ADP, Epi, or PAR4 peptide to activate αIIbβ3. Agonist-elicited soluble fibrinogen binding is therefore thought to be the typical event of inside-out signaling. In this study, the MFI of bound fibrinogen stimulated by ADP/Epi or PAR4 peptide was normalized by that by Mn^2+^ to avoid possible bias derived from the expression efficacy, according to the literature [38]. Our results demonstrated that inside-out signaling was intact in β3-ΔRGT platelets (Fig 3), which was similar to the previous results in CHO cells [5,7,10,32,46] and in the knock-in model [16], indicating that the last three amino acids RGT of the β3 integrin cytoplasmic tail are not indispensable to inside-out signaling. Talin is known to regulate inside-out signaling [5] and kindlin is regarded as a coactivator of talin [8,9], mutations of which also impair the talin-mediated integrin activation [47]. The mutation of the putative binding sites in β3, such as the Y to A substitution at residue 759, causes decreased association with kindlin [18,48] and damaged integrin activation [8]. Our pull-down assays showed that GST-β3-ΔRGT bound talin and kindlin, as did GST-β3, consistent with the intact inside-out signaling in β3-ΔRGT platelets (Figs 6 and 7). In contrast, fibrinogen binding was completely deficient in β3-ΔTNITYRGT platelets (Fig 3), coincident with the previous observation in CHO cells [10]. Pull-down and immunofluorescent results revealed that GST-β3-ΔTNITYRGT bound talin but not kindlin (Figs 6 and 7). These data raised a key question as to whether the TNITY sequence, that contains the conservative NxxY motif, is the core sequence participating in inside-out signaling. A mutant deleting the NITY motif (β3-ΔNITY) was thus designed to address this issue. The fibrinogen binding results showed that, compared to a complete defect of inside-out signaling with the β3-ΔTNITYRGT, the β3-ΔNITY platelets exhibited a partial retention of inside-out signaling (Fig 3). But, as a matter of fact, GST-β3-ΔNITY did not bind kindlin in protein interaction assays, as same as GST-β3-ΔTNITYRGT did (Figs 6 and 7). This might be attributed to a mechanism in which outside-in signaling (preserved in β3-ΔNITY platelets) might give feedback to the regulation of αIIbβ3 activation [49] through other molecules. For instance, vinculin, whose activation status is mediated by outside-in signaling, can increase αIIbβ3 affinity for fibrinogen [50,51].

The αIIbβ3 antagonist RGDS peptide attenuated soluble fibrinogen binding stimulated by ADP/Epi or PAR4 peptide, indicating the specificity of the assay even though the inhibition was incomplete, consistent with previous publications in which the weaker response was attributed to the less sensitivity of rodent platelets to RGDS in comparison to that of human platelets [42,43]. Although potent divalent cation chelating agent, ethylenediamine tetraacetic acid (EDTA), can markedly inhibit the capacity of platelets to bind fibrinogen, it can induce an irreversible change in αIIbβ3 complexes, thus can cause an irreversible loss of their response to agonists [52]. We therefore decided not to use EDTA as an antagonist in the negative control sets for the present study.

Spreading of resting platelets on immobilized fibrinogen surface, probably resulting from the direct interaction of αIIbβ3 with the conformation exposed on immobilized fibrinogen [53,54], is regarded as a typical outside-in signaling independent of inside-out signaling [22] and widely used to assess the outside-in signaling in human platelets [32,55]. The extent of β3^+/-^ platelets to spread was same as that of β3^+/+^ ones (Fig 1C and S5 Fig) suggested that the potential of platelet spreading was not very closely correlated with the expression level of β3 within certain ranges, consistent with the previous publication in which the β3^+/-^ mice have same tail bleeding time, platelet aggregation and clot retraction as β3^+/+^ mice do [31]. Thus the platelet spreading data were not normalized with β3 expression or Mn^2+^-stimulated data. Since mouse platelets spread relatively poorly on immobilized fibrinogen as compared to human platelets, we decided, in addition to the conventional protocols, to observe platelet spreading in the presence of agonists as previously reported in the literature [16,38]. In this case, the contribution of inside-out signals to platelet spreading should be taken into account. Nonetheless, outside-in signaling mediated via the last three amino acids (RGT) is crucial for platelet spreading as β3-ΔRGT with even intact inside-out signaling showed a damaged spreading which are reinforced by the RGT peptide data [6,10]. Our protein interaction data also showed a failure of β3-ΔRGT to bind c-Src (Figs 6 and 7), consistent with the previous study [16]. As expected, the β3-ΔTNITYRGT platelets hardly spread regardless of the presence of agonists (Fig 4), since the putative sequences required for both inside-out or outside-in signaling were deleted and β3-ΔTNITYRGT neither bound kindlin nor c-Src (Figs 6 and 7). The β3-ΔNITY mutant with partially damaged inside-out signaling could spread normally in the absence of agonists (Fig 4), suggesting that the remained RGT motif is sufficient to mediate outside-in signaling and thereby spreading. The association of β3-ΔNITY with c-Src (Figs 6 and 7) also indicated that the RGT sequence is readily interact with c-Src regardless of the existence of the adjacent NITY motif. This is in agreement with our previous work that the only three amino acid sequence, RGT peptide, is able to compete with the endogenous β3 for binding c-Src [32]. Of note, the β3-ΔNITY platelets displayed a partially impaired spreading after ADP and PAR4 peptide stimulation, probably secondary to the partially damaged inside-out signaling pathway. All these results indicated that last three amino acids RGT preserved after deletion of NITY motif were still able to bind c-Src (Figs 6 and 7) and drive the outside-in function, and thus the RGT are not only necessary, but also sufficient for outside-in signaling and adjacent sequences are not required.

Thrombus formation *in vivo* is affected by blood flow and αIIbβ3 signal transduction is required for platelet to resist hemodynamic “washing” [26–28]. There is very few research based on flow assay for transplanted mice because of the dilemma of the small mouse blood volume and the requirement of blood in large quantity to yield high shear rates with conventional *in vitro* perfusion devices. In this study, using the microfluidic devices [29,30], which can provide high shear rates for an appropriate period of time with a requirement of about 0.5 ml blood volume, we tested the adhesion of the platelets bearing different β3 mutants under flow. Two kinds of matrix, fibrinogen and collagen, were used to coat microfluidic channels to explore different mechanisms under flow. The adhesion of unstimulated platelets on fibrinogen-coated surface mainly depends on outside-in signaling [53,54]. The results showed that the ability of platelet adhesion on fibrinogen was in a gradually decreased order from β3, β3-ΔNITY, β3-ΔRGT, to β3-ΔTNITYRGT (Fig 5). Outside-in signaling was more preserved in β3-ΔNITY platelets than in β3-ΔRGT ones, that means, the RGT sequence played more important roles in outside-in signaling than the NITY motif did. Collagen-coated surface can mimic the subendothelial matrix at injured vessels and provide an environment closest to the *in vivo* physiopathological conditions [56], we thus further observed the thrombus formation (adhesion and aggregation) of transfected platelets on collagen-coated surface. The results showed that the extent of adhesion and aggregation of platelets with β3 mutants on collagen progressively decreased from β3-ΔRGT, to β3-ΔNITY, β3-ΔTNITYRGT (Fig 5). On collagen, once the platelets adhere to collagen via the GPVI/α2β1-collagen or GPIb-vWF (recruited by collagen from plasma) interactions, the inside-out signaling starts and the αIIbβ3 will be activated [57]. Then the binding of activated αIIbβ3 to fibrinogen initiates the outside-in signaling which mediates stable adhesion and reversible aggregation. Thus, if the mutations of β3 abolished integrin inside-out signaling, these mutants could also show defective stable adhesion to collagen under flow conditions. Indeed, β3-ΔNITY with impaired inside-out signaling, even with undamaged outside-in signaling, showed a significant decrease in adhesion and aggregation on collagen. However, if a mutation of β3 only affected outside-in signaling such as the β3-ΔRGT, platelets with that mutation showed a partial inhibition of adhesion and aggregation on collagen, indicating that this mutation might still preserve the initial adhesion to collagen, which might mainly help preliminary hemostasis. Recently we also found that *ex vivo* thrombus formation of human platelets under flow was partially inhibited through a selective competition by myr-AC~CRGT peptide with the RGT sequences [58]. When both inside-out and outside-in signaling were impaired in β3-ΔTNITYRGT mutant, adhesion and aggregation on collagen were obviously inhibited. To our knowledge, this is the first observation on the adhesion of mouse platelets with defined defects of outside-in or inside-out signaling under flow.

Calpain cleavage of β3 at the sides of Y^741^, T^747^, F^754^, and Y^759^ occurs progressively from C to N-terminal [15,59,60] during platelet activation, such as aggregation induced by thrombin [59], and spreading on fibrinogen immobilized surface [15]. While cleavage of β3 could limit platelet aggregation, adhesion, and spreading, based on the present study. It is suggested that one of the functional consequences of calpain cleavage of β3 is to negatively regulate platelet function.

In summary, in comparison to an intact inside-out signaling and a markedly inhibited outside-in signaling exhibited by the β3-ΔRGT mutant, β3-ΔTNITYRGT platelets showed obviously impaired bidirectional signaling. In β3-ΔNITY platelets the inside-out signaling pathway was partially affected and outside-in signaling was intact. Under flow, the ability of platelet adhesion was in a gradually decreased order from β3-ΔN^756^ITY^759^, β3-ΔR^760^GT^762^, to β3-ΔT^755^NITYRGT^762^ on fibrinogen-coated surface, or from β3-ΔR^760^GT^762^, β3-ΔN^756^ITY^759^, to β3-ΔT^755^NITYRGT^762^ on collagen-coated surface.

In conclusion, this study showed in platelets that the progressive cleavage of β3 C-terminus by calpain during platelet activation leads to the impairments of platelet function regulated by integrin signals transduced through different directions. The increasing loss of β3 function inhibits the progress of thrombus formation by degrees, which might act as a negative feedback of thrombosis in platelets being stimulated.

## Significance

Upon platelet activation, calpain cleaves the integrin β3 cytoplasmic tail progressively from C-terminus and the pathophysiological outcomes of these cleavages need to be elucidated in platelets. Using a retroviral expression mouse model, platelets bearing the calpain cleavage-related mutations were assayed for their features of signal transduction. Results showed that the T^755^NITYRGT^762^, R^760^GT^762^, and N^756^ITY^759^ sequences participated in bidirectional, outside-in, and inside-out signaling respectively, and thereby regulated thrombus formation under flow. Kindlin and c-Src is involved in the regulation of bidirectional signaling pathways in these processes. The increasing loss of β3 function inhibits the progress of thrombus formation by degrees, which might act as a negative feedback of platelet function in platelets to limit excessive thrombus formation.

## Supporting Information

S1 Fig Representative scatter diagram of fibrinogen binding of transfected platelets with wild-type β3. Fibrinogen binding of the total platelets from a representative transplanted mouse with full-long β3 in the absence (control) or presence of Mn^2+^, ADP/Epi, and PAR4 peptide stimulation. Antagonists (RGDS peptide) were added as an inhibitor. Incomplete inhibition of fibrinogen by RGDS may result from its less sensitivity in rodent platelets than in human platelets.
(TIF)

S2 Fig Genomic DNA fragment was sequenced. The mutated sites were verified in MSCV MigR1 plasmid with wild-type β3 and mutated β3 gene (β3, β3-ΔRGT, β3-ΔTNITYRGT, or β3-ΔNITY).
(TIF)

S3 Fig High expression of GFP in platelets and bone marrow MNCs, but not in RBCs. (A) Peripheral blood (PB) smear and bone marrow (BM) suspension from a representative transplanted mouse under fluorescence, brightfield, and merge of them. (B) GFP expression of platelet (Plt), bone marrow mononuclear cell (MNC), and red blood cell (RBC) from wild-type control or transplanted mouse tested by flow cytometry. (C) Statistical diagram of B. The results are the mean ± SEM from at least five transplanted animals.
(TIF)

S4 Fig The relationship of mean fluorescence intensity of platelet fibrinogen binding stimulated by Mn^2+^ with that of β3 expression. (TIF)

S5 Fig Platelet spreading of β3^-/-^, β3^+/-^, and β3^+/+^ mice on immobilized fibrinogen only (Fg), immobilized fibrinogen accompanied with ADP (Fg+ADP), or PAR4 peptide (Fg+PAR4 peptide). The spreading leave of β3^+/-^ platelets is same as that of β3^+/+^ platelets.
(TIF)

S6 Fig Different mutational β3 and αIIb expressed in the co-transfected 293T cells. (A) Flow cytometric analysis using PE-conjugated anti-human β3 monoclonal antibody showed similar expression levels of β3 among different stably transfected cells. (B) untransfected 239T cells (293T-Vector) and 293T co-transfected cells with β3 and αIIb (293T-β3) were lysed and blotted for SZ21 and SZ22, which recognize the β3 and αIIb, respectively. Actin was used as a loading control. western blot analysis suggested that co-expression of the β3 and αIIb in cells.
(TIF)

S1 Table The blood counts of transplanted mice. Thirty microliter whole blood containing the anticoagulant sodium citrate was collected from transplanted mice, or β3^+/+^, β3^+/-^ and β3^-/-^ mice by cutting tail, then was tested using POCH-100 blood cell counter.
(XLS)

S2 Table Extended data of β3 and GFP expression in the transfected platelets. (XLS)

S3 Table Extended data of fibrinogen binding of transfected platelets. (XLS)

S4 Table Extended data of spreading of transfected platelets on immobilized fibrinogen. (XLS)

S5 Table Extended data of adhesion of transfected platelets under flow. (XLS)
