# Supplementary figures and images for: Functional Effect of the Mutations Similar to the Cleavage during Platelet Activation at Integrin β3 Cytoplasmic Tail when Expressed in Mouse Platelets
Source: PLoS One. 2016 Nov 16;11(11):e0166136. doi: 10.1371/journal.pone.0166136 (PMC5112943; doi:10.1371/journal.pone.0166136)

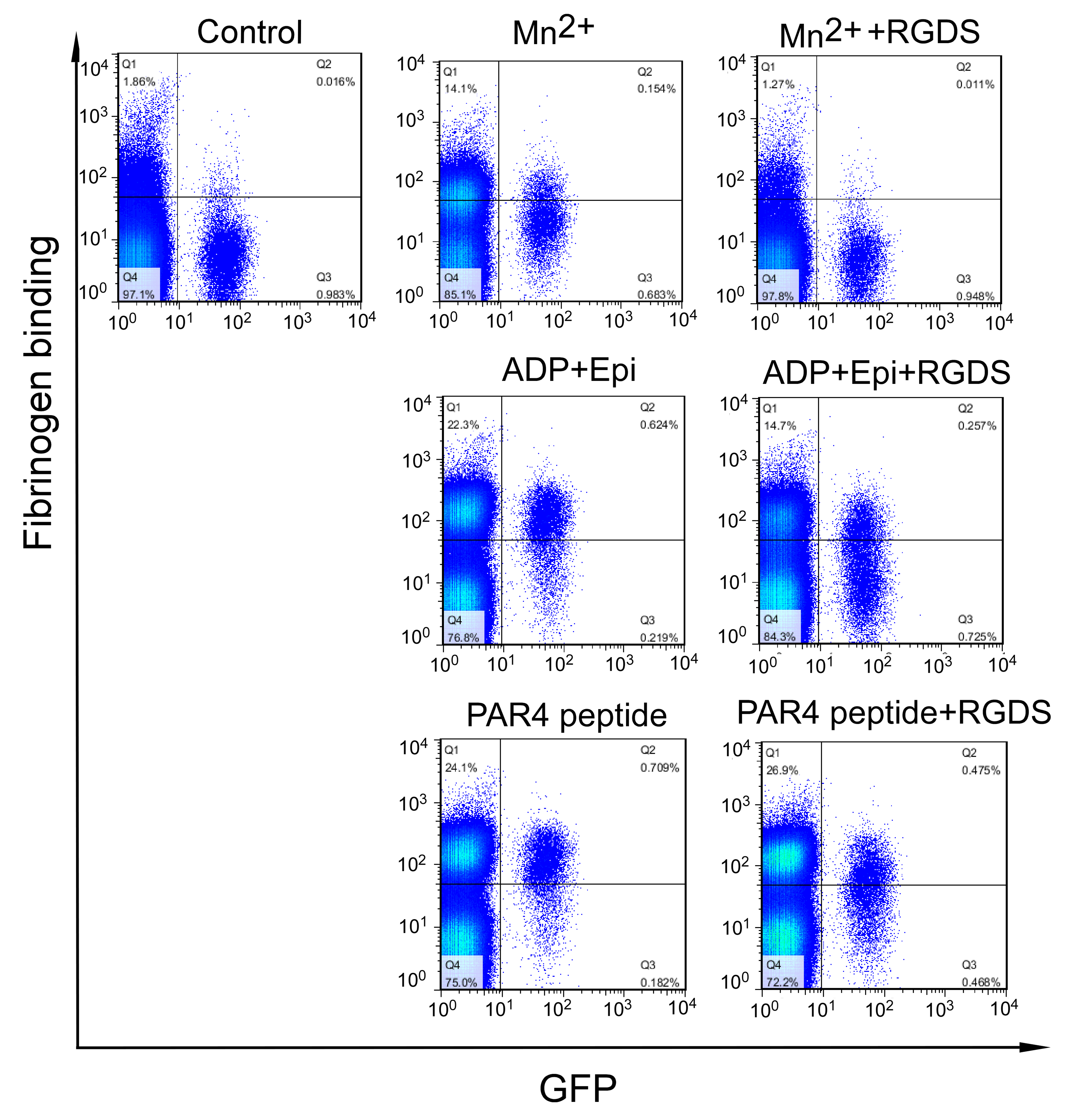

Supplement: S1 Fig — Fibrinogen binding of the total platelets from a representative transplanted mouse with full-long β3 in the absence (control) or presence of Mn2+, ADP/Epi, and PAR4 peptide stimulation. Antagonists (RGDS peptide) were added as an inhibitor. Incomplete inhibition of fibrinogen by RGDS may result from its less sensitivity in rodent platelets than in human platelets. (TIF) [file pone.0166136.s001.tif]

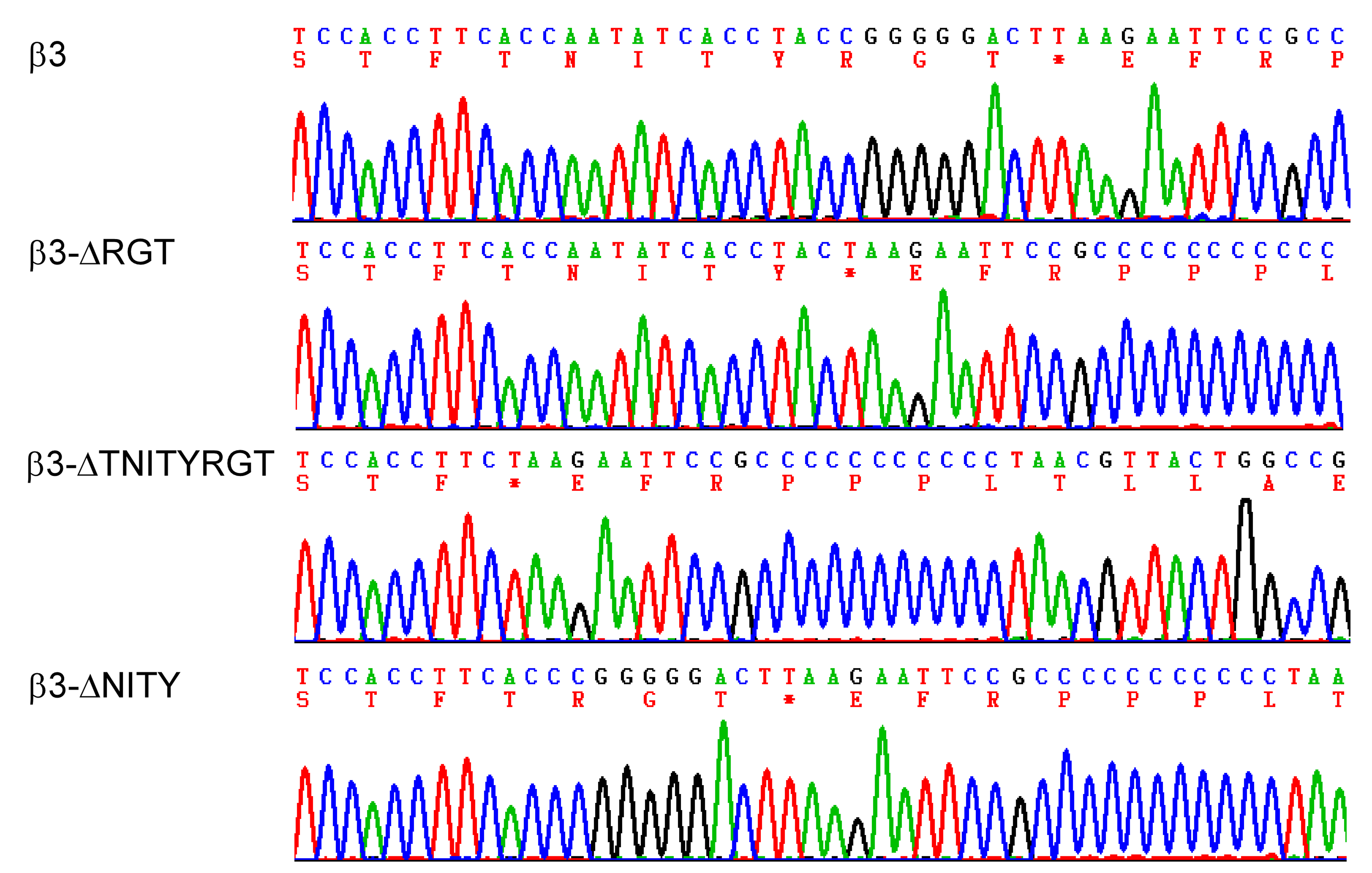

Supplement: S2 Fig — The mutated sites were verified in MSCV MigR1 plasmid with wild-type β3 and mutated β3 gene (β3, β3-ΔRGT, β3-ΔTNITYRGT, or β3-ΔNITY). (TIF) [file pone.0166136.s002.tif]

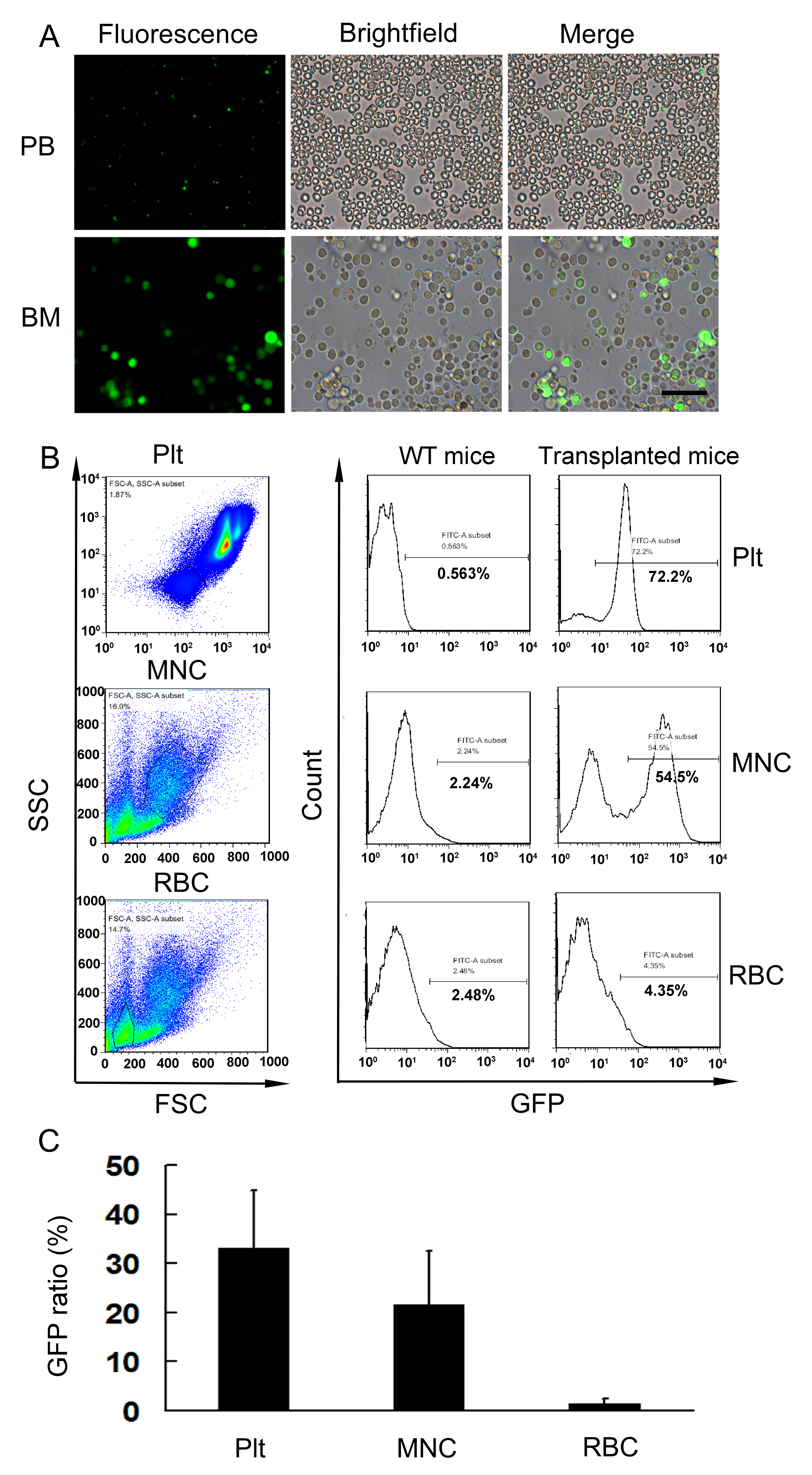

Supplement: S3 Fig — (A) Peripheral blood (PB) smear and bone marrow (BM) suspension from a representative transplanted mouse under fluorescence, brightfield, and merge of them. (B) GFP expression of platelet (Plt), bone marrow mononuclear cell (MNC), and red blood cell (RBC) from wild-type control or transplanted mouse tested by flow cytometry. (C) Statistical diagram of B. The results are the mean ± SEM from at least five transplanted animals. (TIF) [file pone.0166136.s003.tif]

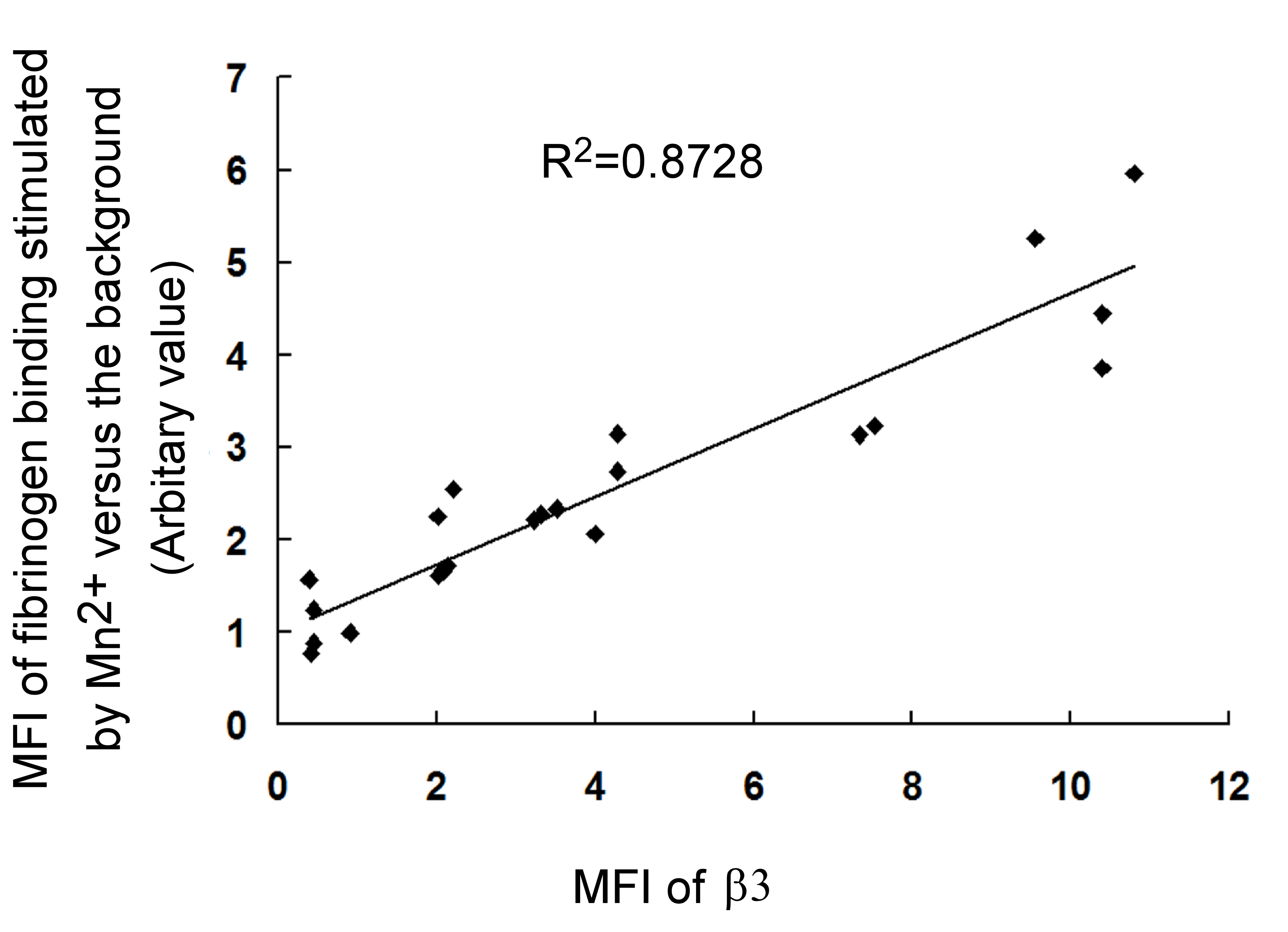

Supplement: S4 Fig — (TIF) [file pone.0166136.s004.tif]

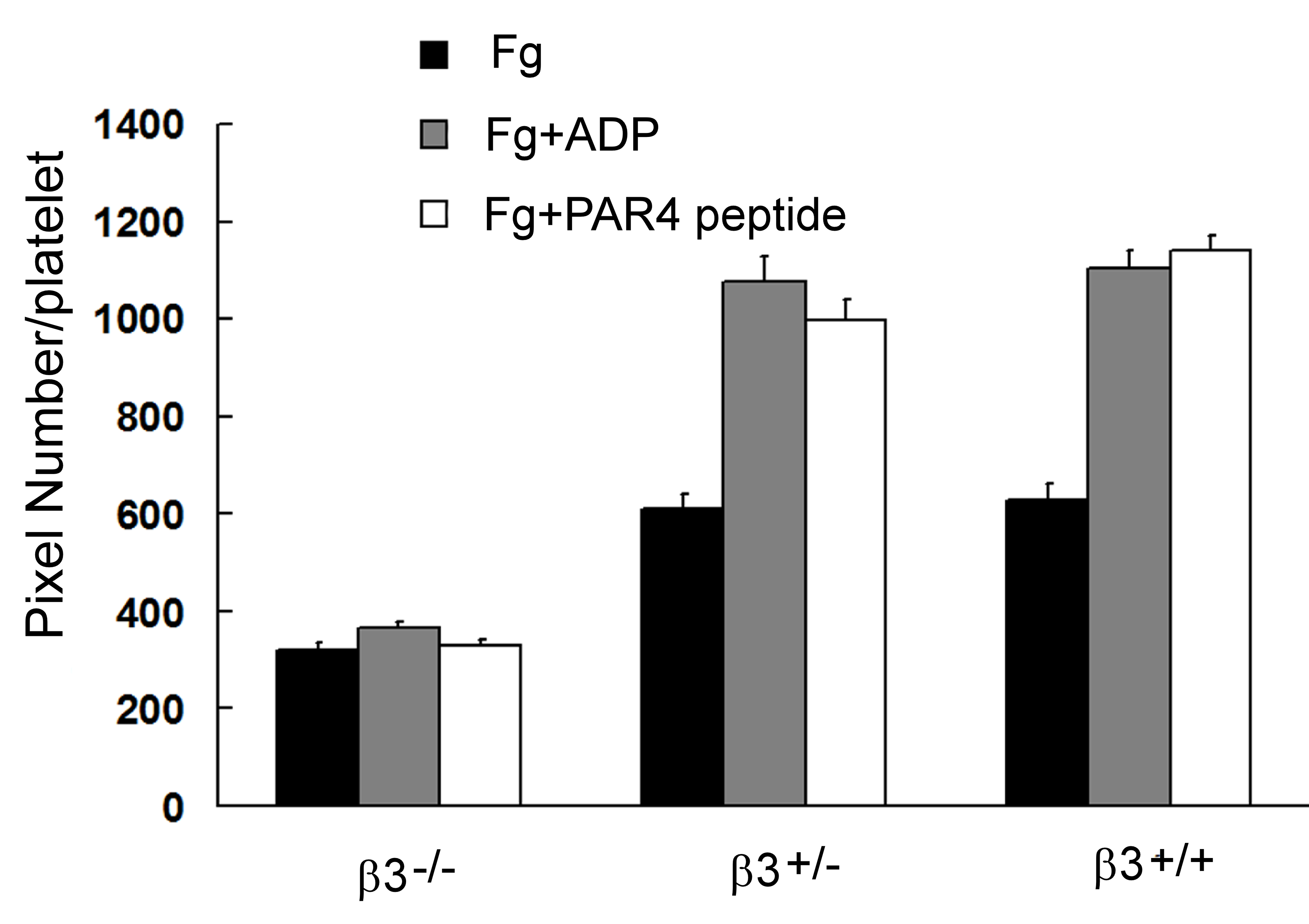

Supplement: S5 Fig — The spreading leave of β3+/- platelets is same as that of β3+/+ platelets. (TIF) [file pone.0166136.s005.tif]

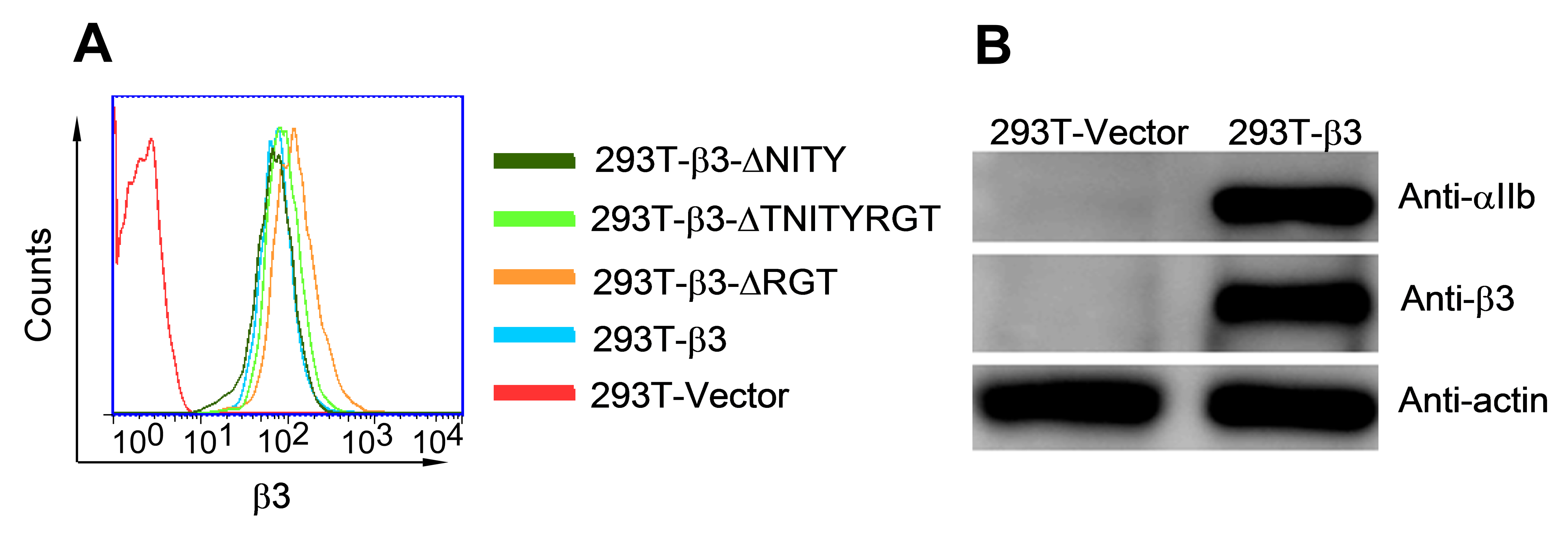

Supplement: S6 Fig — (A) Flow cytometric analysis using PE-conjugated anti-human β3 monoclonal antibody showed similar expression levels of β3 among different stably transfected cells. (B) untransfected 239T cells (293T-Vector) and 293T co-transfected cells with β3 and αIIb (293T-β3) were lysed and blotted for SZ21 and SZ22, which recognize the β3 and αIIb, respectively. Actin was used as a loading control. western blot analysis suggested that co-expression of the β3 and αIIb in cells. (TIF) [file pone.0166136.s006.tif]
